# Situational Awareness and Health Protective Responses to Pandemic Influenza A (H1N1) in Hong Kong: A Cross-Sectional Study

**DOI:** 10.1371/journal.pone.0013350

**Published:** 2010-10-12

**Authors:** Qiuyan Liao, Benjamin Cowling, Wing Tak Lam, Man Wai Ng, Richard Fielding

**Affiliations:** 1 Health Behaviour Research Group, Department of Community Medicine, School of Public Health, The University of Hong Kong, Hong Kong Special Administrative Region, China; 2 Infectious Diseases Group, Department of Community Medicine, School of Public Health, The University of Hong Kong, Hong Kong Special Administrative Region, China; Singapore Immunology Network, Singapore

## Abstract

**Background:**

Whether information sources influence health protective behaviours during influenza pandemics or other emerging infectious disease epidemics is uncertain.

**Methodology:**

Data from cross-sectional telephone interviews of 1,001 Hong Kong adults in June, 2009 were tested against theory and data-derived hypothesized associations between trust in (formal/informal) information, understanding, self-efficacy, perceived susceptibility and worry, and hand hygiene and social distancing using Structural Equation Modelling with multigroup comparisons.

**Principal Findings:**

Trust in formal (government/media) information about influenza was associated with greater reported understanding of A/H1N1 cause (β = 0.36) and A/H1N1 prevention self-efficacy (β = 0.25), which in turn were associated with more hand hygiene (β = 0.19 and β = 0.23, respectively). Trust in informal (interpersonal) information was negatively associated with perceived personal A/H1N1 susceptibility (β = −0.21), which was negatively associated with perceived self-efficacy (β = −0.42) but positively associated with influenza worry (β = 0.44). Trust in informal information was positively associated with influenza worry (β = 0.16) which was in turn associated with greater social distancing (β = 0.36). Multigroup comparisons showed gender differences regarding paths from trust in formal information to understanding of A/H1N1 cause, trust in informal information to understanding of A/H1N1 cause, and understanding of A/H1N1 cause to perceived self-efficacy.

**Conclusions/Significance:**

Trust in government/media information was more strongly associated with greater self-efficacy and handwashing, whereas trust in informal information was strongly associated with perceived health threat and avoidance behaviour. Risk communication should consider the effect of gender differences.

## Introduction

Pandemic influenza A/H1N1 has a clinical profile similar to seasonal influenza, despite initially appearing more severe [1]. Respiratory infectious diseases (RIDs) such as influenza are a major public health issue best dealt with by prevention, ideally vaccination. However, in the first six-months or so of a newly-emergent RID epidemic/pandemic vaccines are generally unavailable and non-pharmacological interventions can play a major role in minimizing RID spread [2]–[4]. Government health education messages are a major source of information for promoting self-protective practices against RIDs. These preventive messages generally emphasize improved hygiene, face-mask use by infected persons, and social distancing measures, including avoiding crowds during epidemics [5]–[7].

**Figure 1:**
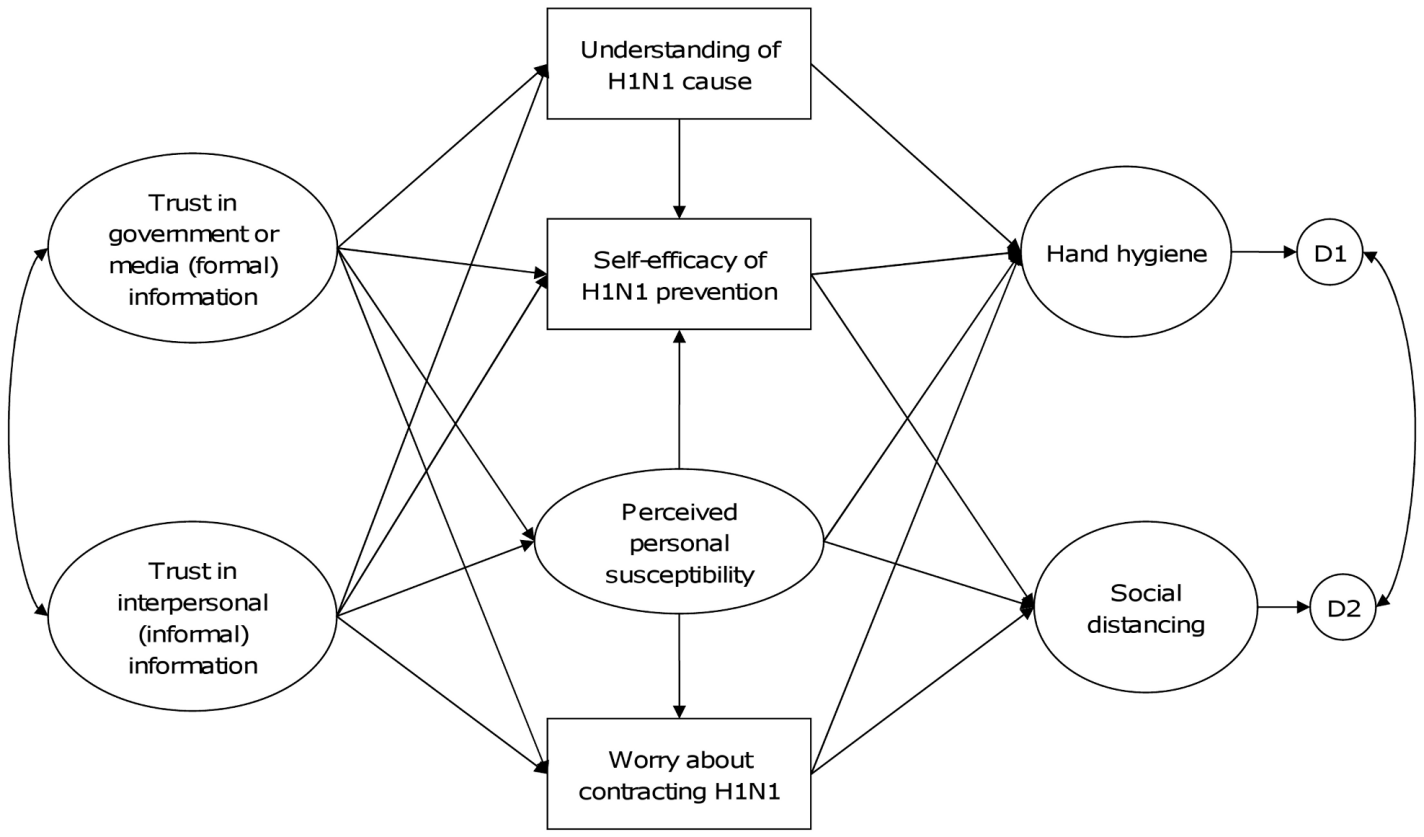
A hypothesized model for health protection against pandemic influenza A/H1N1.

Predictors of population uptake of health protective behaviours in RID epidemics have begun to be studied [8]–[13], yet related theory remains nascent and this is problematic: to effectively predict behaviour during future epidemics robust theory is critical. Effective models that enable comprehensive prediction of health protective behaviours remain limited mainly to two overlapping theoretical paradigms: the Theories of Reasoned Action/Planned Behaviour (TPB) [14]–[16] and Bandura's concept of self-efficacy [17]–[19] (the belief that one can successfully execute some behaviour), particularly regarding the core TPB concept of perceived behavioural control, which controversially is claimed by some to be largely synonymous with self-efficacy [19]–[21] and by others to be indistinguishable from intent [22] (the intention to execute a particular behaviour), the key predictive element of TPB [16]. When used to account for health-related behaviours TPB-based models typically account for ∼35% of variance in outcomes [16], while self-efficacy accounts for ∼25% of variance in outcomes [23], [24]. However, neither TPB nor Self-efficacy allow for the social and affective influences that might be expected logically to be important in RID [25], [26]. We report on a theoretical model that incorporated elements of influenza causal knowledge, perceived self-efficacy and also social and affective influences (Figure 1) because these latter variables have been less frequently studied in combination, but have theoretical and logical support for their potential importance in the context of RIDs. We tested this model against data collected in the early phase of the influenza A/H1N1 pandemic (Table S1) to examine how levels of trust in formal and informal sources of risk/prevention information associated with hand washing and social distancing.

## Methods

### Ethics Statement

Ethics approval was obtained from the Institutional Review Board of the University of Hong Kong/Hospital Authority Hong Kong West Cluster. For this telephone interview, written informed consent was waived by the IRB but verbal consent was required from all the respondents and agreement to participate in the interview was taken as further consent. Before the interview began, a brief introduction about the study aims and interview contents was given and then respondents were asked whether the interview could start. If approval was received this was recorded and the interview performed. If not, respondents were thanked and the call was terminated.

### Sampling

More than 98% of Hong Kong households have landline telephones and all local calls are free. Random-digit dialled telephone numbers and within-household random-sampling grids (Kish grids) are a cost-effective way to survey highly representative random population samples. Kish grids are matrices containing random numbers for different sized households that facilitate random selection of individuals within households and help minimize sampling bias. The number of eligible household residents, “n”, is determined by asking the person of first contact in the household. The Kish grid provides a randomly generated number “k” between 1 and “n” which is used by the interviewer. Ordering by age and starting from the oldest eligible member in the household, the k'th member is then invited to participate in the survey. Different grid values are used for each household. As part of a series of surveys to monitor A/H1N1 epidemic activity, a commercial polling organization administered the questionnaire using this telephone-survey methodology, targeting 1,000–1,500 participants on each occasion, a sample size calculated to give an estimate of A/H1N1 health protective behaviours with a precision of ±3%. The survey with the largest sample was selected for this analysis. Sampling was performed during the evening to minimize exclusion of young working adults.

### Sources of data

Data on attitudes, knowledge, situational awareness, risk perception and preventive behaviours (Table S1) were collected by household telephone interviews, based on random digit dialling. One Cantonese-speaking adult (age≥18) who lives >4 nights per week in each household was selected using a Kish grid. All interviews were conducted between 8:30pm–10:45pm from 23^rd^–25^th^ June, 2009, two weeks after the first community transmission had been identified in Hong Kong.

### Behavioural model

Existing theoretical frameworks of behaviour change have been adapted to predict health-related behaviour-change for chronic, non-communicable diseases [15], [16], but we lack a comprehensive evidence-based model of protective behaviour against RID threat [11]. A recent review of 26 papers on RID prevention behaviours concluded that 23 lacked a theoretical basis [13]. Existing applications of health behaviour change models in communicable disease are almost exclusively limited to HIV/AIDS research [24], [27] and to a lesser extent hepatitis B and C, which share the same transmission pathways as HIV. There are good reasons why sexually-transmitted diseases embody a different set of influences than do RIDs. For example people are highly motivated to seek sexual contact (or injection drug use) and have a high degree of potential control (e.g. condom use) over the nature of these encounters, even though they may be situationally constrained from executing that control, and are infected only by direct exchange of bodily fluids. In contrast, one can acquire an RID transmitted by air droplets, hand contact or fomites for up to 72 hours after the person who is the source departs [28], or immediately by being sneezed on. Infection is much more casual. Clearly, the controllability of RIDs requires different behavioural imperatives to those in STDs and hence different psychological influences should be considered. Attempts by the TPB to accommodate social influences had relied on incorporating social norms [14], the behavioural expectations within a group. However, norms, and hence theoretical models reliant on norms to account for social processes, cannot accommodate the fact that communicable respiratory diseases make other humans ambiguous sources of threat: one can usually control sexual encounters but not who shares public transport. In this respect social factors in communicable respiratory disease differ significantly from those in non-communicable diseases and warrant greater consideration than existing HBC models allow. Outbreaks of new infectious diseases constitute situations that are uncertain, dynamic, and embody highly personal threat, requiring rapid decisions on appropriate action [29]. Under such circumstances timely and relevant information on the best preventive actions become critical to such decision-making. Hence, health protective behaviour during the early stages of a novel epidemic would be more likely to resemble situational reactions using established or known default actions such as avoiding crowds (social distancing), rather than intention-based planning before any behavioural change, such as deciding to consult a doctor to administer a vaccination. Later in the epidemic as threat familiarity increases, different factors such as planned behaviour may become important.

Reporting delays, uncertainty and other biases affect publicly available information on the characteristics of newly-emergent communicable diseases, such as A/H1N1 lay knowledge of infection-related risks can be limited. The resulting uncertainty about disease severity and transmissibility at the epidemic onset extends to the utility and timing of adopting preventive measures. Information cues to individuals about initiating protective action must therefore be synthesized from various sources. Perceived information reliability or trustworthiness influences decisions to utilize any given information source [30] to inform awareness of the situation. More trustworthy sources are therefore likely to be more influential. Epidemic situational awareness is likely derived from formally-announced public information like news items, government press releases and health education messages, and also from informal, social sources [25], [29]; observation of other peoples' behaviour and communications from family, peers and neighbours. Noting how others behave informs action decisions in the observer [19]. If those around you are wearing masks, this indicates others might have knowledge you do not possess, and that the threat level might be locally high and imminent, suggesting prudent precautionary or RID preventive behaviour. Observers are also subject to social conformity influences that can help adoption of group patterns of behaviour. Maintaining situational awareness, involving elements of perception, comprehension and prediction [31], during epidemics probably relies on these two types of information. However, when uncertainty is high and widespread, or when there is low confidence in social and other information sources then individuals' HPBs might be expected to be more independent of formal and informal information sources.

Perceived risk is influenced by several stimulus characteristics, including unfamiliarity, invisibility, dreadfulness and inequity [32], and by recipient characteristics, including demographics and trust in information source and content [33]. Perceived risk is an important determinant of protective behavioural responses [12], [34], [35], [36], but is subject to optimistic bias, where for example people distort their risk of contracting influenza downwards relative to others [35], [37]. Nonetheless, susceptibility to risk remains an important measure in understanding variation in behavioural responses to threat and reflects the key element of perceived risk in an epidemic/pandemic situation. Worry is a cognitive process linked to anxiety [26], [38] and reflects negative affectivity, interacting with perception of susceptibility to risk [26], [39] and may also influence RID protective responses such as social distancing [13].

### Measures

Because data were collected using telephone interviews we had to adapt measures to suit a brief format in order to avoid people hanging up mid-way or providing invalid answers to hurry the interview, a problem encountered with this data collection method. We therefore used parsimonious measure to minimize assessment fatigue and low response rates which threaten representativeness.

Trust in government/media (formal) information: We asked about respondents' agreement with three statements (Table S1). Responses were made on categorical five-point scales ranging from “strongly disagree” to “strongly agree”. Scalability of these three items was assessed using Cronbach's alpha, which at 0.61 indicated that the internal consistency between items was low, but acceptable. However, to minimize potential measurement error arising from the low internal consistency, this construct was treated as a latent variable in the subsequent analysis [40]. A latent variable is a concept opposed to an observed variable. A latent variable can not be measured directly but is inferred from one or more variables that are directly measured (observed variable) while an observed variable can be directly measured with a specific question or item or observed by the researchers. For example, an “attitude” is a concept that is difficult to measure directly with single items but can be inferred from various questions asking about different aspects of that attitude. Then within the analysis “attitude” is treated as the latent variable while the questions used to infer it are the observed variables.

Trust in interpersonal (informal) information: Respondents' agreement with two statements (Table S1). Responses were made on categorical five-point scales ranging from “strongly disagree” to “strongly agree”. Scalability of these two items was assessed using Cronbach's alpha, which at 0.50 indicated that scalabilty was unsuitably low for two items. This suggests that these two items measure different aspects of social information. Again to minimize potential measurement error this construct was treated as a latent variable in the subsequent analysis.

Understanding cause of A/H1N1 (“I understand how Swine flu is caused”) and self-efficacy (confidence in one's ability to act in a way that achieves desired future outcomes) for A/H1N1 prevention (“I am confident that I can protect myself against Swine flu”): Each was assessed using responses on 5-point scales of agreement with these two single item statements (Table S1).

Perceived personal susceptibility: Two items, one assessing absolute susceptibility (perceived absolute probability of developing A/H1N1) and another assessing relative susceptibility (perceived probability of developing A/H1N1 relative to peers) formed a latent variable for perceived personal susceptibility (Table S1). The Cronbach alpha of these two items was 0.66.

Worry about contracting H1N1. Respondents were asked to indicate their level of worry over the past one week about contracting influenza A/H1N1. Responses were 5-point scales of worry ranged from “never thought about it” to “extremely worried” (Table S1).

Hand hygiene. Respondents were asked to indicate frequencies of use of four hand hygiene practices over the three days prior to interview: hand washing after sneezing, coughing and touching nose; hand washing after returning home, use of liquid soap for hand washing, and hand washing after touching common objects. Responses were on a 4-point scale of frequency: 1 “never”, 2 “sometimes”, 3 “usually” and 4 “always”. Cronbach's α was 0.62 (Table S1).

Social distancing behaviours: a. Social Avoidance. Respondents were asked to indicate if they had adopted any of four avoidance behaviours due to influenza A/H1N1 in the past 7 days: avoiding eating out, avoiding using public transport, avoiding going to crowded places, and rescheduling travel plans Responses were coded as 1 “yes” and 0 “no”. Cronbach's α was 0.61 (Table S1).

### Statistical analysis

We first compared the demographic structure of the sample against that of the general population derived from the Hong Kong government General Household Survey to identify any sample differences.

Our model proposes that trust in formal (government and media sources) and informal (from other people) information affects RID epidemic health protective behaviours, the former by informing about generic risk and response characteristics for dealing with a potential threat (causes and protective responses), the latter about threat imminence, severity and response effectiveness (seeing how others behave). We refer to the product of these combined processes as situational awareness, and propose that rather than driving behaviour directly information acts through altering the cognitive/affective domain of situational awareness. Thus the model is predicated on several premises: that understanding of the disease and perceived personal susceptibility influence self-efficacy [17], [18], [31]; that the effect of perceived susceptibility to influenza on HPBs acts through increasing worry about the disease [26], [33], [38], [41], [42]; and that more worry from perceived susceptibility prompts HPBs [39], [41], [42]. These cognitive/affective processes are represented in the hypothesized model (Figure 1).

Structural Equation Modeling (SEM) is a method for simulating and testing multiple and interrelated causal relationships simultaneously in statistical data, making it suitable for theory development and testing [40]. SEM was applied to test the hypothesized model. SEM is usually performed when a model contains latent variables assessed with specified measurement models. Despite including estimations of a series of multiple regression equations, SEM differs from regression analysis in several ways, which make it advantageous for this kind of analysis. First, SEM is usually theoretically based because it is performed after researchers specify the hypothesized model. Second, it can be used to refine the hypothesized model by estimating the measurement model and structural model simultaneously. Finally SEM analysis can accommodate measurement errors of the constructs in the model [40].

In our hypothesized model, trust in formal information, trust in informal information, perceived personal susceptibility, hand washing and social distancing behaviours were entered as latent (inferred) variables while other constructs were entered as observable (directly measured) variables because they were assessed with only one item. Two different health protective behaviors, hand washing and social distancing, were entered as the HPB outcomes because we hypothesized that different influences may act on each of these. We assumed that the “disturbances” of the two health behavior outcomes were correlated. Disturbance represents the unexplained variances of the latent variables predicted by the specified independent variables [40]. In making this assumption, we assumed that unexplained variance in the outcome variables could be correlated and the variables in question jointly influenced by other unknown factors, and so allowed for such constraints within the model by using more conservative criteria. Previous studies have shown that hand hygiene and social distancing behaviours during a pandemic could be influenced by some common causes which were not fully explored in our study such as current health, past experience of disease and cues to action [14]. In particular, in our study, the two kinds of health protective behaviours occurred in the same situation of the 2009 influenza pandemic, and so it is sensible and reasonable to assume that they could be influenced by some common causes which were not fully explored in our studies.

Adequacy of the measurement models was tested before testing the full structural model. To test the full structural model, all constructs (Figure 1) were entered into the model and all factor loading, specified paths, covariance, measurement errors and disturbances were estimated simultaneously. Since the model contained categorical variables, Weighted Least Square with mean- and variance-adjusted estimation (WLSMV) was used to estimate the standardized parameter (β) for each path [43]. With this kind of estimation, chi-square difference testing is inappropriate. We therefore used the Comparative Fit Index (CFI), Tucker-Lewis Index (TLI) and Root Mean Squared Error of Association (RMSEA) to evaluate the model fit to the data. A CFI>0.95, TLI>0.95 and RMSEA<0.05 indicate a good fit of data to the model [43]. The analysis was conducted in Mplus 6.0 for Windows [43]. The proportion of missing values ranged from 0.1% for “In the past one week, have you ever worried about catching influenza A/H1N1” to 10.1% “did you wash hands after sneezing, coughing or touching nose in the past 3 days”. Missing data were handled with multiple imputation to generate 10 datasets which were summarized into one for subsequent analysis. Multiple imputation was performed in AmeliaView [44].

Responses are likely to differ by sociodemographic factors [13]. We therefore stratified the sample by gender and by age (<45 years old vs. >45 years old). Education is also likely to have a significant effect but there are difficulties in education stratification in Hong Kong. The age cut-off of 45 years was adopted to account for the introduction in Hong Kong of 6-year compulsory education in 1971 and 9-year compulsory education in 1978 [45]. This means that people aged 45 or above are much less likely to have a tertiary (college/university) level education and less secondary (high school) education than people aged <45 years old [45]. Moreover, in traditional families in China, a son (who lived with his parents after marriage) was usually more educationally-favoured over daughters (who moved to their in-laws' home on marriage) to ensure support for the parents in their old age, so males usually obtained more education than females [45]. These distinctions were somewhat evidenced by our data which showed that 98% of the respondents aged <45 compared to 71% of the respondents aged 45 or above (χ^2^ = 147.69, p<0.001), and 89% of male compared to 80% of female respondents, obtained at least secondary education (χ^2^ = 17.05, p<0.001). Since the numbers of tertiary educated respondents and primary (elementary) educated respondents were too small to produce stable models, we limited stratification to gender and age only and acknowledge that this also incorporates indefinable education and income effects.

Consequently, we used a multi-group SEM to assess the invariance of the model (Figure 1) across gender and age group (respondents aged 18–44 and aged 45 or above). We tried to test the model by stratifying the sample into four subgroups (female aged 18–44, female aged 45 or above, male aged 18–44 and male aged 45 or above). However, the sample size for males aged 18–44 was relative small (Table S2). Moreover, all the model variables were treated as categorical variables and we used the WLSMV method to estimate the model. This method requires that each subsample covers all the categories of each variable. In the case of one category, younger males, not all variable values were present. To meet the assumptions for analysis we would need to recode all variables, intrinsically altering the model. In order to avoid this, we relinquished a combined four-group comparison and instead compared the model across gender and the two age groups separately. To perform multi-group comparison we first ran a model with all parameters unconstrained. We then identified factor loadings that were not significantly different (p≥0.05) and set these as equal, while loadings that were significantly different were allowed to vary, and finally paths that did not differ significantly were constrained to be equal while those that differed significantly were allowed to vary and estimated separately by groups. The “DIFFTEST” option in MPlus 6.0 was used to obtain a correct chi-square difference test for the WLSMV estimators and was used to estimate the differences between the least constrained model (with all the paths freely estimated) and the most constrained model (with all the paths constrained to be equal) as well as the partially constrained model (with some of the paths freely estimated and others constrained to be equal) [43]. A p-value>0.05 for the “DIFFTEST” indicate a non-significant difference between the models.

Finally, to help interpret these multigroup SEM comparisons, we performed a post-hoc examination of the model variable means for different gender and age groups and tested differences using the Mann-Whitney test, which tests differences between two groups on ordinal scales of measurements.

## Results

A total of 1,001/1,449 (69.1% response rate) Hong Kong adults successfully completed the interview. The characteristics of the sample were compared against the Hong Kong 2006 by-census population data [46], showing respondents to be better educated and more likely to have been born in Hong Kong compared to the general population (Table 1) but otherwise representative.

**Table 1:** Characteristics of the sample compared with the Hong Kong population.

Characteristics	Sample	Population structure^a^	Effect size^b^
Age group			
18–34 years	29.1%	33.1%	0.09
35–54 years	41.8%	41.2%	
≥55 years	28.4%	25.7%	
Gender			
Female	53.7%	52.3%	0.03
Male	46.3%	47.7%	
Marital status			
Single	31.4%	32.4%	0.02
Married/formerly married	67.3%	67.6%	
Education			
Primary or below	15.8%	25.4%	0.30
Secondary	49.7%	51.6%	
Tertiary or above	33.9%	23%	
Birth place			
Hong Kong	68.0%	60.3%	0.16
Other places	31.5%	39.7%	

a Based on 2006 Hong Kong by-census (Census & Statistics Department HKSAR).

b Effect sizes 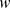 are calculated via the formula 
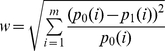
, where 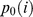 and 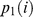 are the observed proportions in the i'th category from the by-census data and survey data, respectively.

Both formal and informal information trust were correlated with all situational awareness variables except worry about contracting A/H1N1 (“Worry”), while formal information trust was also independent of perceived personal susceptibility (“Susceptibility”). In turn, understanding of H1N1 cause (“Understanding”) and Perceived self-efficacy (“Self-efficacy”) were significantly associated with hand washing while Worry and Susceptibility were significantly associated with social distancing (Table S3).

**Figure 2:**
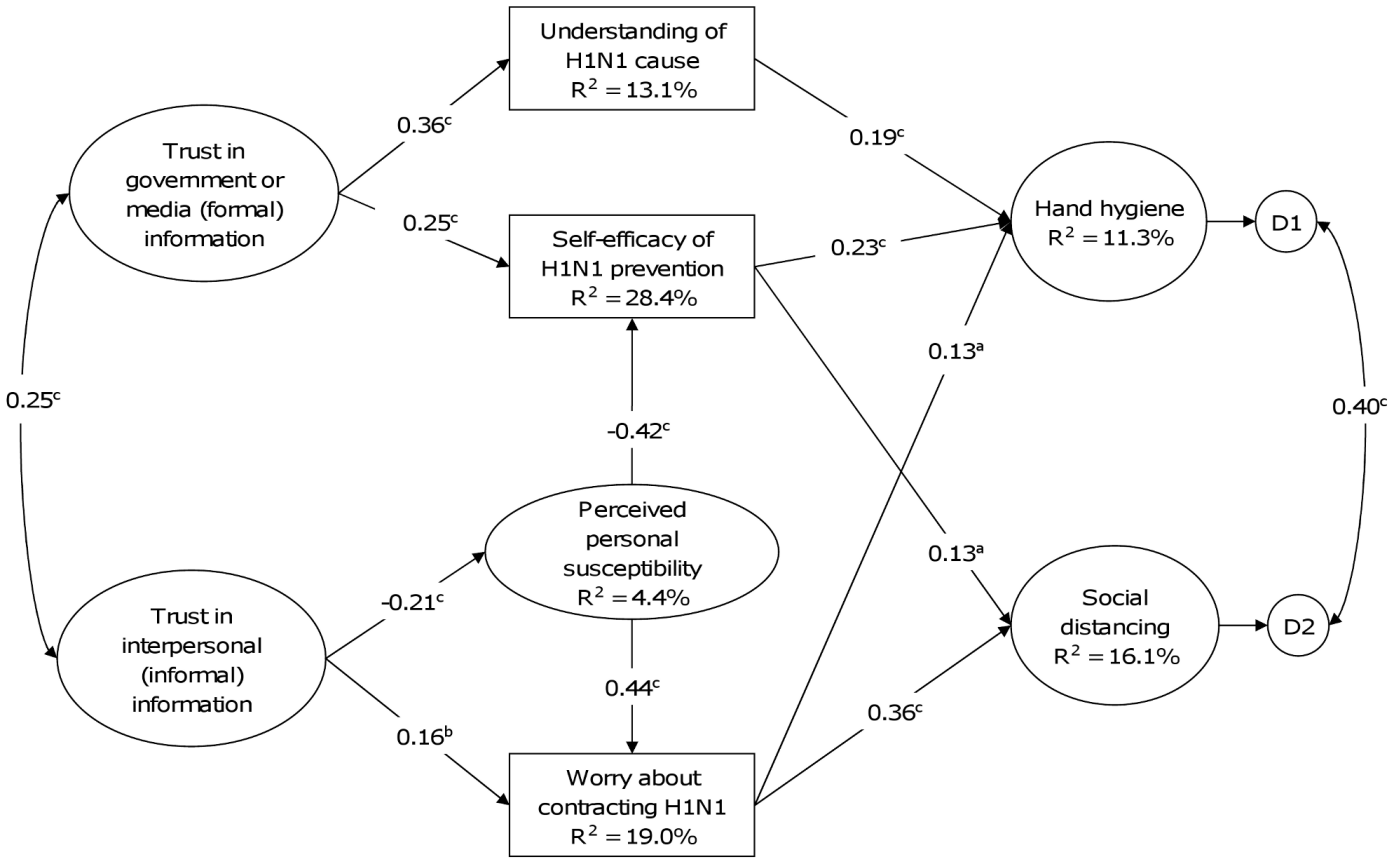
Structural equation model for health protection against the 2009 influenza pandemic among Hong Kong general people. N (the sample size) = 1001. The numbers represent standardized path coefficients (β). Only paths with statistically significant (p<0.05) β≥0.1 are included. Significant paths with β<0.1 were considered trivial and excluded to aid clarity. ^a^ p<0.05, ^b^ p<0.01, ^c^ p<0.001.

The SEM model fitted well to the data with CFI = 0.977, TLI = 0.969 and RMSEA = 0.026. Standardized coefficients indicated two primary features in the model; the first one linking Formal information and hand hygiene and a second linking Informal information and Social distancing (Figure 2). Paths were seen via Formal information trust and Self-efficacy (β = 0.25) and Self-efficacy and hand hygiene (β = 0.23), and via Formal information trust and Understanding (β = 0.36), and Understanding and hand hygiene (β = 0.19) while Understanding and Self-efficacy were independent. These associations formed the first feature. Marginal associations between Worry and hand hygiene and between Self-efficacy and social distancing were seen, but the small standardized coefficients of β = 0.13 suggest that these paths are minor. Susceptibility and Worry were associated, but otherwise were functionally independent, both upstream from formal information trust, and downstream from hand hygiene.

The second feature of the model is reflected in a different set of paths associating informal information trust with social distancing. Trust in informal information sources was inversely associated with Susceptibility (β = −0.21), which was associated positively with Worry (β = 0.44), and inversely with Self-efficacy (β = −0.42). However, more confidence in informal information sources was associated with more Worry (β = 0.16) and finally, only Worry was associated with social distancing (β = 0.36). Trust in informal information was independent of Understanding and Self-efficacy.

The only remaining notable feature of the model was a strong inverse association (β = −0.42) between Susceptibility and Self-efficacy. This suggests some interaction between these two variables that could strongly influence both sets of paths mentioned so far.

Overall, the model explained 11.3% of the variance in hand hygiene and 16.1% of the variance in social distancing behaviors.

### Multiple group comparison

**Figure 3:**
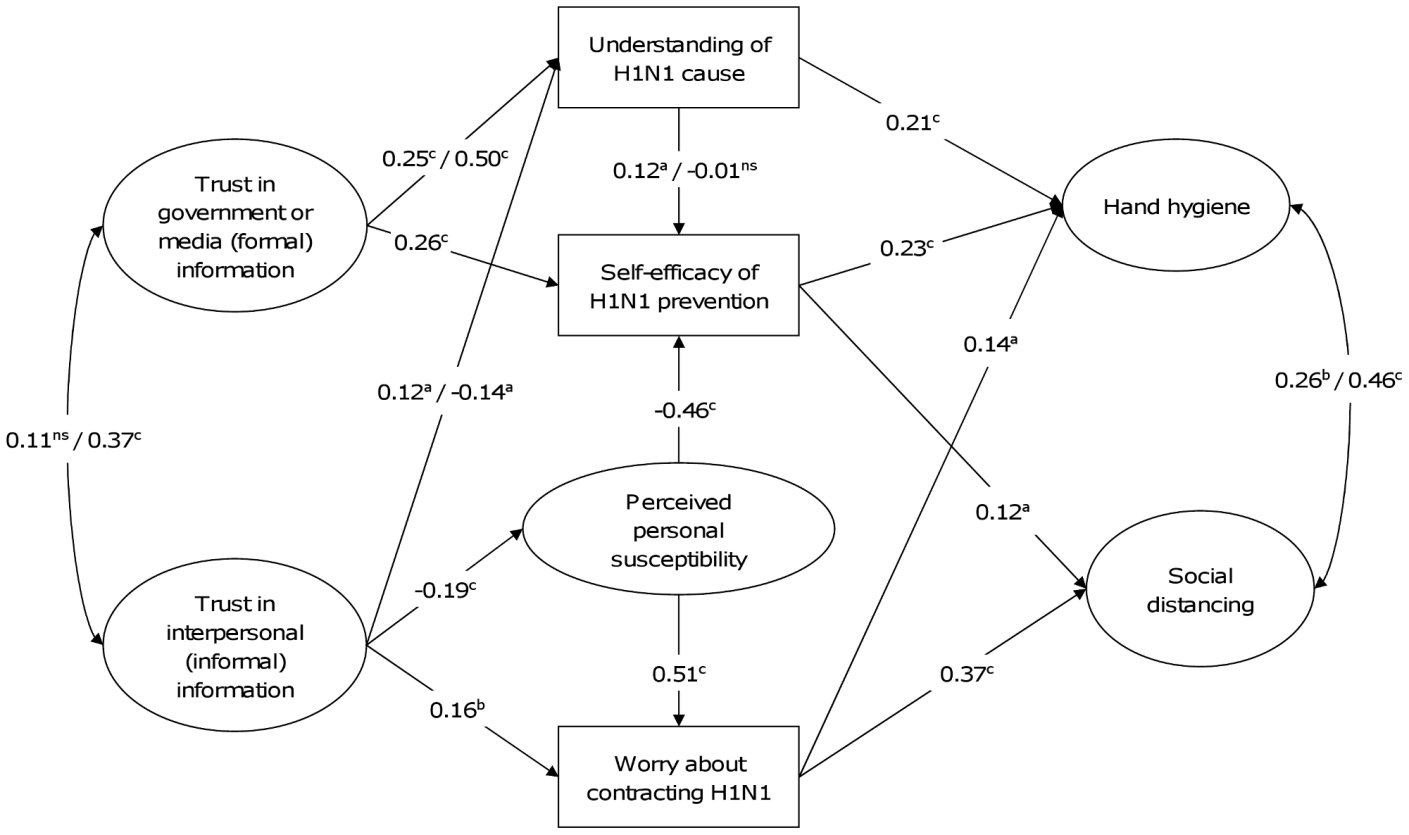
Multigroup comparison of the structural equation model for health protection against the 2009 influenza pandemic applied to males and females. The numbers represent standardized path coefficients (β). For a given path, if the path coefficients did not differ significantly between males and females, only the path coefficient for males is presented; if the path coefficients differed significantly between males and females, the path coefficients for both genders are presented with the ones for males presented on the left of the slashes and the ones for female presented on the right of the slashes. ^a^ p<0.05, ^b^ p<0.01, ^c^ p<0.001.

Across gender, both the least constrained model and the most constrained models fit the data well with CFI>0.970, TLI≥0.970, RMSEA = 0.025. The most constrained model did not differ significantly from the least constrained model (χ^2^ for “DIFFTEST” = 29.30, d_f_ = 19, p = 0.061). However, three sets of associations differed significantly between females and males: those between Formal information trust and Understanding, from Informal information trust to Understanding, and from Understanding to Self-efficacy. These paths were set free and estimated separately in female and male. The model with these paths freely estimated fit well to the data with CFI = 0.978, TLI = 0.976 and RMSEA = 0.023, and did not differ significantly from the least constrained model (χ^2^ for “DIFFTEST” = 15.07, d_f_ = 16, p = 0.519). Figure 3 presents the results of multigroup comparison of the model applied to males and females with the three path parameters unconstrained. For a given path, if the path coefficients did not differ significantly between males and females, only the path coefficient for males is presented; if the path coefficients differed significantly between males and females, the path coefficients for both genders are presented with the coefficients for males presented on the left of the slashes and for females presented on the right of the slashes.

By comparison, the model shows that for both genders while the association between Formal information trust and Understanding was positive this association was stronger amongst females (β = 0.50) than males (β = 0.25); the association between Informal information trust and Understanding was weakly positive in males (β = 0.12) but weakly negative in females (β = −0.14), and; the association between Understanding and Self-efficacy was positive (β = 0.12) in males but non-significant in females (β = −0.01).

Across the two age groups, both the least constrained model and the most constrained model fit well to the data with CFI>0.960, TLI≥0.950, RMSEA≤0.030. The most constrained model did not differ significantly from the least constrained model (χ^2^ for “DIFFTEST” = 15.85, d_f_ = 19, p = 0.667). No path was found to be significantly different between the two age groups.

Means and standard deviations for all model variables by gender and age group showed differences (Table S4). All the constructs did not differ by gender except for hand hygiene and social distancing with female being more likely to wash their hands and adopt social distancing behaviours. Trust in formal and informal information sources, Self-efficacy, and Hand hygiene significantly differed by age groups, with respondents of older age group being more likely to trust the information from both sources, perceive higher self-efficacy and wash their hands.

## Discussion

We tested a hypothesized model of associations between trust in (formal/informal) information, situational awareness variables (causal understanding, self-efficacy, susceptibility and worry) and different types of health protective behaviours (hand hygiene and social distancing) for influenza protection. The model suggested that two different sets of influences relate trust in information to hand hygiene, and to social distancing respectively. The strongest associations observed were between Susceptibility and Self-efficacy (negative), Susceptibility and Worry (positive), Trust in Formal information and Understanding (positive), Trust in Formal information and Self-efficacy (positive), and Trust in Informal information and Susceptibility (negative), and Worry and social distancing (positive).

### Trust in Formal information

Neither age nor gender contributed significant variation to the association between Trust in Formal information and Self-efficacy, and Self-efficacy and hand hygiene. These findings are consistent with other studies showing self-efficacy is enhanced by procedural information [18], [19], [47], [48] and that attitudinally and action-oriented interventions are more successful in changing behaviour for communicable disease protection, such as in the case of HIV [24]. Similarly, exposure to relevant media stories during the 2009 A/H1N1influenza pandemic was associated with higher efficacy beliefs regarding hygiene, which in turn was associated with greater frequency of reported tissue access and sanitising gel purchase among British people [49]. However, there is evidence that coping style interacts with the ability of procedural information to enhance self-efficacy and under circumstances of high threat, such as during SARS-type epidemics where mortality is high, procedural information might be counter productive for some segments of the community who use an information avoidance (“blunting”) coping style [50]. Self-efficacy was only weakly associated with social distancing. People are limited in their ability to avoid crowds in Hong Kong, one of the most densely populated cities on earth, despite the Hong Kong government recommending this in order to limit the pandemic [51]. However, the relatively mild impact of A/H1N1 meant that people saw no reason to jeopardize their economic well-being and curtail other social activities, given such a low perceived threat [49], [52]. Hand washing was probably seen as sufficient protection.

The association between Trust in Formal information and Understanding of influenza cause differed by gender but not age, with females showing a stronger association. Men tend to have poorer health knowledge than women [53]. We found that females were more likely to wash their hands than were males. Older respondents reported significantly greater trust in formal information, marginally-significantly better understanding of influenza cause and were more likely to wash their hands. This is consistent with other studies reflecting that preventive practice is enabled by knowledge of causes [49], [54]. However, increasing knowledge is not itself sufficient to always ensure preventive behaviour [55]. In this context, Understanding has an independent contribution to hand washing practice only.

### Trust in Informal information

Trust in Informal information seems to be associated with less perceived susceptibility to health threat. This may reflect rational processes or cognitive bias. Trusting social cues involves comparison and conformity influences, and can enhance optimistic bias (the tendency to view oneself less likely to experience negative events but more likely to experience positive events) in personal risk estimates [56], thereby reducing perceived Susceptibility. Conversely, others' behavioural cues about health threat proximity can arouse motivating worry and anxiety producing protective action [17],[29]. We found Trust in informal information was independent of both Understanding of influenza cause and Self-efficacy. However, when stratified by gender, the Trust in informal information-Understanding association was positive among males but negative among females. Education is probably an important influence in understanding and may have a bearing on these patterns which await clarification.

Susceptibility was strongly associated with both Self-efficacy (negatively) and Worry (positively). Neither Worry nor Susceptibility varied significantly by gender or age group. This is plausible and theoretically consistent [26], [34], [35], [39]. Worry was strongly associated with social distancing, again consistent with British data [49]. Although Worry was also significantly associated with hand hygiene, the association was weak. Elsewhere, using a generic measure of personal hygiene practices we have found a stronger association between disease worry and hygiene, suggesting a moderate effect of level of disease worry [57].

The model tested explained only a modest proportion of the variance in adoption of HBPs, suggesting that there are significant theoretical gaps that remain to be filled. These await further research.

### Social distancing

Social distancing is unassociated with formal HPB messages, suggesting potential susceptibility to a “herd-like” response in this Chinese community, particularly if confidence in formal (government or doctors) information is low. Voerten and colleagues describe such a pattern of response in the early stages of SARS [25]. These models support the hypothesis that social distancing is more likely to occur when perceived health threat is high [25]. Logically, when others seem to be behaving in a way that is informed and probably consistent then their actions provide clear information. If mixed social messages occur signalling uncertainty then the utility of social information will fall. This is likely to be associated with increase perceived susceptibility, and possibly greater worry and distancing behaviour. This pattern of responses would be most likely early in a novel RID epidemic where disease characteristics and behaviour are often uncertain. High threat uncertainty then drives social avoidance of potentially high-risk others. High levels of worry are associated with greater social distancing. Around 50% of 997/14,297 (response rate 7%) British respondents agreed that social avoidance would minimize risk of A/H1N1 infection, and respondents reporting more anxiety were more likely to engage in preventive actions; severity and likelihood of infection were the most important determinants of preventive action [12]. Further research on social influences on HPB during epidemic and pandemic RIDs is warranted. Providing more knowledge about disease causes can improve hand hygiene but is unlikely to influence social avoidance, which appears less amenable to formal health messages. However, as formal messages achieve acceptance across the population, and uptake of HPBs increases, then under circumstances where a critical mass of the population are practicing precautions trust in informal information should increase, reducing susceptibility and worry and leading to declines in social avoidance. Because others are likely adopting HPBs this makes them less of a contagion risk. Conversely maintaining a high level of hand washing practices may require sustained public education activities. Finally, different segments of the population probably communicate different types of information with their peers.

Self-efficacy in preventing A/H1N1 influences hand hygiene but has little influence on social distancing. Formal health education messages that focus on enhancing the public's sense of their ability to protect themselves by adopting hygiene practices would seem to be the most effective to improve hand hygiene, but where the practice is already established, high levels of trust in these messages are not likely to significantly increase hand hygiene.

### Limitations

This study is limited in being cross-sectional and relying on hypothesized modeling to infer causality. This is potentially error-prone and can only be confirmed by specific longitudinal tests of the hypotheses proposed above. There are potential limitations related to measurement imposed by the need to be parsimonious in questioning due to use of telephone interviews. Where this is not done refusal rates would have been unacceptably high [12] raising serious questions about representativeness. As a consequence, construct validity for some latent variables was weaker than expected, for example, only two items were scaled to measured trust in informal information giving a low internal consistency. We re-ran the SEM treating the two trust items as separate which gave almost identical associations with different situation awareness variables, so we entered their combined score as a latent variable in the final model. Only one item measured self-efficacy. This is generally not considered adequate but does have precedent indicating it is valid for predicting behavioral change [9]. Finally, this random sample, closely representative of the population of Hong Kong and collected early in the epidemic phase, nonetheless was slightly older and less-well educated than the general population. This was likely due to unavoidable sampling bias from surveying in the early evening to 10pm. Many young adults do not return home from work until after this time and were thereby not sampled. The results may in part reflect this bias. Otherwise the response rate was high at 69% and excellent compared to similar studies [12]. Some of these above limitations may also have contributed to the low explained variance of the model.

### Implications

Many factors influence RID protective behaviour. This study has examined a very limited number of these. Confidence in formal information such as health education messages is associated with greater compliance to recommended preventive measures for influenza A/H1N1 [12]. However, the mechanisms for this were unclear. We have shown that this probably involves different mechanisms for hand washing and social distancing, and suggest how these might function. Formal messages may not reduce social distancing behaviours until such time that preventive behaviours are widely adopted in the community. Social distancing seems more likely to occur when there is high influenza-related worry and uncertainty, such as in the initial stages when epidemic circumstances are unknown, or if an epidemic is severe and appears poorly controlled, as during early SARS. This would seem to be largely worry/affect-driven. If so, then social distancing is likely to occur irrespective of government messages as population anxiety about an epidemic increases. Susceptibility may also increase and this may inhibit self-efficacy regarding hand washing. Finally, high levels of community uncertainty or rumour are likely to increase distancing by exacerbating perceived susceptibility and worry.

A simple version of our findings can be found it the supporting file (Text S1).

## Supporting Information

Table S1 (0.05 MB DOC)

Table S2 (0.03 MB DOC)

Table S3 (0.04 MB DOC)

Table S4 (0.04 MB DOC)

Text S1 This is a simple version of our study findings for nonspecialists.
(0.03 MB DOC)
