# Supplementary material for: Situational Awareness and Health Protective Responses to Pandemic Influenza A (H1N1) in Hong Kong: A Cross-Sectional Study
Source: PLoS One. 2010 Oct 12;5(10):e13350. doi: 10.1371/journal.pone.0013350 (PMC2953514; doi:10.1371/journal.pone.0013350)
Supplement: Table S1 — (0.05 MB DOC) [file pone.0013350.s001.doc]

Table S1. Overview of the measures

| Measures | Items | Scale | Mean (SD) | α |
| --- | --- | --- | --- | --- |
| Trust in government or media information | I am persuaded by what I read in the paper about swine flu | 1-5 | 3.78 (0.73) | 0.61 |
|  | Media reports about swine flu can be trusted | 1-5 | 3.81 (0.82) |  |
|  | I trust what the government says about swine flu | 1-5 | 3.65 (0.85) |  |
| Trust in interpersonal communication | The best source of information about swine flu is to watch others and listen to what they say | 1-5 | 2.60 (1.07) | 0.50 |
|  | I tend to believe what my friends, colleagues or neighbors say about swine flu rather than the papers or TV news | 1-5 | 2.40 (0.94) |  |
| Understanding of H1N1 transmission | I understand how people get infected with swine flu | 1-5 | 3.58 (0.94) | - |
| Efficacy belief of H1N1 prevention | I am confident that I can prevent myself from catching swine flu | 1-5 | 3.53 (1.04) | - |
| Perceived personal susceptibility | How likely do you think it is that you will contracting swine flu over the next 1 month | 1-7 | 3.41 (1.17) | 0.66 |
|  | How likely is it that you will contract swine flu compared with other people of your group? | 1-7 | 3.10 (1.16) |  |
| Worry about contracting H1N1 | In the past one week, have you ever worried about catching influenza A/H1N1 | 1-5 | 1.54 (0.86) | - |
| Hand hygiene (in the past 3 days) | Wash hands after sneezing, coughing or touching nose | 1-4 | 2.99 (1.05) | 0.62 |
|  | Wash hands after returning home | 1-4 | 3.48 (0.89) |  |
|  | Use liquid soap when washing hands | 1-4 | 3.28 (1.04) |  |
|  | Wash hands after touching common objects (e.g., door knobs, lift button) | 1-4 | 2.33 (1.25) |  |
| Social distancing behaviour (in the past 7 days) | Avoid eating out due to swine flu | 0-1 | 0.10 (0.30) | 0.61 |
|  | Avoid using public transport due to swine flu | 0-1 | 0.05 (0.22) |  |
|  | Avoid going to crowded places due to swine flu | 0-1 | 0.30 (0.46) |  |
|  | Reschedule travel plan | 0-1 | 0.19 (0.39) |  |
