# Supplementary material for: Situational Awareness and Health Protective Responses to Pandemic Influenza A (H1N1) in Hong Kong: A Cross-Sectional Study
Source: PLoS One. 2010 Oct 12;5(10):e13350. doi: 10.1371/journal.pone.0013350 (PMC2953514; doi:10.1371/journal.pone.0013350)
Supplement: Table S2 — (0.03 MB DOC) [file pone.0013350.s002.doc]

Table S2, Sample size of repartition of males and females in the two age groups (aged 18-44 and aged 45 or above)

| Gender | Age group | |
| --- | --- | --- |
|  | Aged 18-44 | Aged 45 or above |
| Female | 277 (47%) | 316 (53%) |
| Male | 184 (46%) | 217 (54%) |
| Total | 461 (46%) | 533 (53%) |
