# Supplementary material for: Situational Awareness and Health Protective Responses to Pandemic Influenza A (H1N1) in Hong Kong: A Cross-Sectional Study
Source: PLoS One. 2010 Oct 12;5(10):e13350. doi: 10.1371/journal.pone.0013350 (PMC2953514; doi:10.1371/journal.pone.0013350)
Supplement: Table S3 — (0.04 MB DOC) [file pone.0013350.s003.doc]

Table S3. Correlation matrix between the measures

| Measures | 1 | 2 | 3 | 4 | 5 | 6 | 7 | 8 |
| --- | --- | --- | --- | --- | --- | --- | --- | --- |
| 1. Influence of government or media information | 1 |  |  |  |  |  |  |  |
| 2. Influence of interpersonal communication | .165^b^ | 1 |  |  |  |  |  |  |
| 3. Understanding of H1N1 transmission | .237^b^ | .097^b^ | 1 |  |  |  |  |  |
| 4. Efficacy belief of H1N1 prevention | .204^b^ | .171^b^ | .119^b^ | 1 |  |  |  |  |
| 5. Perceived personal susceptibility | -.043 | -.146^b^ | .026 | -.334^b^ | 1 |  |  |  |
| 6. Worry about contracting H1N1 | -.031 | .024 | -.041 | -.110^b^ | .260^b^ | 1 |  |  |
| 7. Hand hygiene | .108^b^ | .040 | .149^b^ | .185^b^ | -.049 | .058 | 1 |  |
| 8. Social distancing behaviour | .053 | .045 | .055 | .007 | .106^b^ | .239^b^ | .236^b^ | 1 |

^a^ p<0.05, ^b^ p<0.01
