# Supplementary material for: Situational Awareness and Health Protective Responses to Pandemic Influenza A (H1N1) in Hong Kong: A Cross-Sectional Study
Source: PLoS One. 2010 Oct 12;5(10):e13350. doi: 10.1371/journal.pone.0013350 (PMC2953514; doi:10.1371/journal.pone.0013350)
Supplement: Table S4 — (0.04 MB DOC) [file pone.0013350.s004.doc]

Table S4. Mean (Standard Deviation) and differences of model constructs by gender and age groups

|  | Gender | | p^e^ | Age group | | p^e^ |
| --- | --- | --- | --- | --- | --- | --- |
| Constructs | Female | Male |  | Aged 18-44 | Aged 45 or above |  |
| Trust in formal information ^a^ | 3.77 (0.57) | 3.70 (0.64) | 0.172 | 3.69 (0.61) | 3.79 (0.59) | 0.004 |
| Trust in informal information ^a^ | 2.52 (0.81) | 2.47 (0.84) | 0.424 | 2.37 (0.77) | 2.61 (0.84) | <0.001 |
| Understanding of H1N1 cause ^a^ | 3.57 (0.92) | 3.59 (0.98) | 0.731 | 3.54 (0.91) | 3.61 (0.97) | 0.055 |
| Perceived self-efficacy ^a^ | 3.51 (1.04) | 3.55 (1.04) | 0.612 | 3.46 (1.02) | 3.58 (1.06) | 0.038 |
| Perceived personal susceptibility ^b^ | 3.26 (0.95) | 3.24 (1.01) | 0.972 | 3.31 (0.99) | 3.20 (1.01) | 0.070 |
| Worry about contracting H1N1^a^ | 1.56 (0.91) | 1.50 (0.78) | 0.655 | 1.58 (0.89) | 1.50 (0.83) | 0.145 |
| Hand washing^c^ | 3.11 (0.70) | 2.88 (0.73) | <0.001 | 2.96 (0.74) | 3.06 (0.71) | 0.044 |
| Social distancing behaviour^d^ | 0.73 (0.99) | 0.50 (0.85) | <0.001 | 0.62 (0.93) | 0.65 (0.95) | 0.527 |

^a^ Mean score ranging from 1-5; ^b^ Mean score ranging from 1-7; ^c^ Mean score ranging from 1-4; ^d^ Total score ranging from 0-4.

^e^ p-value for group differences based on Mann-Whitney test.
