# Supplementary material for: Situational Awareness and Health Protective Responses to Pandemic Influenza A (H1N1) in Hong Kong: A Cross-Sectional Study
Source: PLoS One. 2010 Oct 12;5(10):e13350. doi: 10.1371/journal.pone.0013350 (PMC2953514; doi:10.1371/journal.pone.0013350)
Supplement: Text S1 — This is a simple version of our study findings for nonspecialists. (0.03 MB DOC) [file pone.0013350.s005.doc]

**Extended summary**

We applied statistical modelling to test a theoretical framework that might explain differences in hand washing and avoiding other people, (preventive behaviours adopted during the A/H1N1 influenza pandemic) on data from a sample of 1,001 people interviewed by telephone in Hong Kong. We explored the question: does trust of the news about influenza and trust in what people encountered in daily life say and do about influenza possibly influence thoughts and feelings that previous studies have linked to health protective activities? The theoretical framework fitted the data well. We compared the model when applied to females and males, as well as to younger and older people, and found some gender differences. Higher trust in what other people say and do linked more in male but less in female to the understanding of influenza’s cause. Better understanding of influenza’s cause linked to higher confidence in one’s ability to prevent A/H1N1 pandemic influenza in males but not in females. These results provide very specific findings that are consistent with the more general reports in the current literature.
